# Feasibility and Tolerability of Daily Microgreen Consumption in Healthy Middle-Aged/Older Adults: A Randomized, Open-Label, Controlled Crossover Trial

**DOI:** 10.3390/nu17030467

**Published:** 2025-01-28

**Authors:** Sylvia Y. Lee, Kiri A. Michell, Michelle M. Butler, Brayden T. Smith, Emily K. Woolf, Sydney C. Holmes, Lauren E. Grabos, Allegra R. Vazquez, Hanan Isweiri, Marisa Bunning, Mark E. Uchanski, Sangeeta Rao, Steven E. Newman, Tiffany L. Weir, Sarah A. Johnson

**Affiliations:** 1 Department of Food Science and Human Nutrition, Colorado State University, Fort Collins, CO 80523, USA; sylvia.lee@colostate.edu (S.Y.L.); kiri.michell18@alumni.colostate.edu (K.A.M.); emily.woolf@pbrc.edu (E.K.W.); tiffany.weir@colostate.edu (T.L.W.); 2 Pennington Biomedical Research Center, Baton Rouge, LA 70808, USA; 3 Department of Horticulture and Landscape Architecture, Colorado State University, Fort Collins, CO 80523, USAmark.uchanski@colostate.edu (M.E.U.);; 4 Department of Biology, University of Benghazi, Benghazi 16063, Libya; 5 Department of Clinical Sciences, Colorado State University, Fort Collins, CO 80523, USA; sangeeta.rao@colostate.edu

**Keywords:** anthocyanins, betalains, cardiovascular, glucosinolates, gut, phytochemicals, human health, nutritional quality, sustainability

## Abstract

**Background/Objectives:** Microgreens are rich in nutrients and phytochemicals that can support healthy aging, including attenuation of cardiovascular disease risk. The nutrient and phytochemical contents of red beet (i.e., bull’s blood’ beet, *Beta vulgaris*) and red cabbage (*Brassica oleracea var capitate*) microgreens, as well as existing preclinical evidence suggest their cardioprotective effects, but the feasibility, gastrointestinal tolerability, and human health effects of daily microgreen consumption are unknown. This study aimed to evaluate the feasibility and gastrointestinal tolerability of 2 weeks of daily microgreen consumption in healthy middle-aged/older (MA/O) adults. A secondary aim was to characterize potential health effects. **Methods:** Healthy MA/O adults (initial n = 26) were randomized to consume either 2 cups of ‘bull’s blood’ beet or red cabbage microgreens daily for 2 weeks in a crossover design, with each treatment period separated by 2 weeks. Feasibility was determined through participant retention and intervention compliance (i.e., total doses consumed divided by 14 days), while gastrointestinal tolerability was determined by a gastrointestinal health questionnaire and bowel movement log. Impacts of microgreen consumption on brachial and aortic hemodynamic parameters, and gut microbiota composition were evaluated. **Results:** Daily consumption for 2 weeks of ‘bull’s blood’ beet and red cabbage microgreens was found to be feasible as indicated by high participant retention (final n = 24) and overall treatment compliance of 95.6%. Gastrointestinal symptom severity was not impacted overall, though an improvement in gastrointestinal inflammation-associated symptom severity scores following the red cabbage microgreen intervention (*p* = 0.047) was observed. There were no changes in bowel movement quality, hemodynamic parameters, or on alpha or beta diversity of the gut microbiota. **Conclusions:** Daily consumption of ‘bull’s blood’ beet and red cabbage microgreens is feasible and tolerable in healthy MA/O adults. Future studies designed to evaluate their health impacts are needed.

## 1. Introduction

The world population is projected to reach 9.7 billion in 2050 and 10.4 billion in 2100 [1]. The rapidly growing population is leading to increased urbanization and adverse impacts on environmental health and natural resources, which has important implications for the global food supply and human health [2,3]. Likewise, the prevalence and burden of chronic disease is rapidly increasing worldwide [4]. Chronic diseases such as cardiovascular disease (CVD) stem from complex interactions including intrinsic risk factors (e.g., aging, genetics) and external risk factors (e.g., environment, behavior) [5,6]. As the global population and life expectancy increases, the prevalence and burden of chronic disease will follow. Thus, multi-targeted and multidisciplinary approaches are needed to ensure a nutritious, safe, accessible, and sustainable food supply to support human and environmental health.

Microgreens are an emerging, but underutilized, horticultural food crop with high potential to promote human health while preserving environmental resources [7]. Distinct from sprouts and baby greens, microgreens are the edible, young and tender cotyledonary leafy greens of most vegetables, herbs, and grains, and some flowers [8]. They are grown indoors in greenhouses, using controlled environment agriculture (e.g., vertical farming), and in other environments such as restaurants and homes [8,9]. Depending on the species, microgreens are harvested approximately 10–20 days after sowing at the emergence of the plant’s first true leaves. Previous research has demonstrated their high nutritional quality and contents of phytochemicals linked to human health, and comparisons with mature plant counterparts (e.g., mature vegetables) have shown that many (but not all) of these compounds are higher in microgreens [10,11]. For example, red cabbage microgreens have been shown to contain more vitamin C, beta-carotene, calcium, and magnesium than mature red cabbage, while ‘bull’s blood’ beet microgreens have more vitamin C, vitamin K, vitamin E, and beta-carotene than raw red beets [12,13,14]. We previously demonstrated that red cabbage and ‘bull’s blood’ beet microgreens contain higher polyphenol contents than mature counterparts [11]. These microgreens can be an excellent source of essential nutrients; for example, a 2-cup serving of red cabbage microgreens can provide over half of the Recommended Daily Allowance (RDA) of vitamin C for women [14]. Importantly, microgreen production can have a reduced environmental footprint due to factors such as lower water requirements and reduced transport during distribution within the food system [7].

We and others have previously shown that microgreens are acceptable to consumers (i.e., high acceptability/liking overall, and for appearance, texture, and flavor), though certain factors such as sensory perception and food neophobia (i.e., reluctance to try new or unfamiliar foods) may impact their acceptability [15,16,17]. Microgreens are popular in culinary establishments as garnishes due to their diverse colors, flavors, and textures, which can enhance the organoleptic properties and enjoyment of meals. However, their potential for widespread applications and benefits spanning agriculture, human health, and economic and environmental sustainability suggests that they could be better utilized as food crops. To date, few studies have explored their potential implications for human health [18,19,20] and while promising, the feasibility and tolerability of daily microgreen consumption has not been evaluated in humans. Furthermore, the potential health implications of ‘bull’s blood’ beet microgreen or red cabbage microgreen consumption in humans are unknown.

The primary objective of this study was to investigate the feasibility of daily microgreen consumption in healthy middle-aged/older (MA/O) adults, a population inherently at risk for chronic disease development, namely CVD. Secondary objectives were to explore potential impacts on gastrointestinal tolerability, hemodynamic parameters, gut microbiota, and diet quality. We hypothesized that daily microgreen consumption for 2 weeks would be feasible and not associated with adverse gastrointestinal symptoms. Due to the nature of feasibility studies and the baseline health status of the participants, no major impacts were expected on health-related outcomes.

## 2. Materials and Methods

### 2.1. Participants

Healthy MA/O men and postmenopausal women (menopausal status was self-reported as ≥1 y absence of menses) aged 45 to 70 years with a BMI between 18.5 and 29.9 kg/m^2^ were recruited to participate in this randomized, open-label, controlled crossover trial. Exclusion criteria determined at the in-person screening visit included diagnosed CVD, hypertension, diabetes, neuropathy, thrombosis, cancer, gastrointestinal, kidney, liver, lung, or pancreatic disease, taking medication for blood lipids, blood pressure, or glucose control, or hormone therapy medications, blood pressure ≥ 130/80 mmHg, triglyceride levels > 200 mg/dL, low density lipoprotein cholesterol levels ≥ 160 mg/dL, total cholesterol levels ≥ 240 mg/dL, and/or hemoglobin A1c ≥ 6.5%, a weight change ≥ 3 kg in the past 3 months, actively trying to lose weight, unwilling to maintain their normal dietary or physical activity patterns throughout the study, smoking cigarettes or have a history of smoking cigarettes in the past 12 months, taking antibiotics or have taken them within 1 month of the study, binge and/or heavy drinkers (>3 drinks on any given occasion and/or >7 drinks/week for women, or >4 drinks on any given occasion and/or >14 drinks/week for men), allergies or contraindication to study foods, procedures, or procedure supplies.

### 2.2. Participant Recruitment

Participants were recruited from the greater Fort Collins, Colorado area through Colorado State University (CSU) email correspondence and paper flyer distribution between July and November 2019. Prospective participants sent an email or called the Food and Nutrition Clinical Research Laboratory at CSU to indicate interest and were then asked a series of questions regarding their health and medical history to determine eligibility through a phone prescreening. Those meeting eligibility criteria through the prescreening process were invited for an onsite screening visit at the clinical research laboratory where they provided written informed consent, and inclusion and exclusion criteria were confirmed through a series of assessments. This included obtaining a detailed health history from the participant, followed by a 10-min seated rest in a quiet room prior to blood pressure assessment (measured in triplicate with 1-min intervals and averaged) using an automatic device (Omron Healthcare, Inc., Kyoto, Japan). A blood draw was performed to assess lipid profiles (Piccolo Xpress Chemistry Analyzer, Abbott, Abbott Park, IL, USA) and hemoglobin A1c (Alere Afinion Analyzer System, Abbott). Measurement of height, weight, waist and hip circumferences were performed.

**Figure 1:**
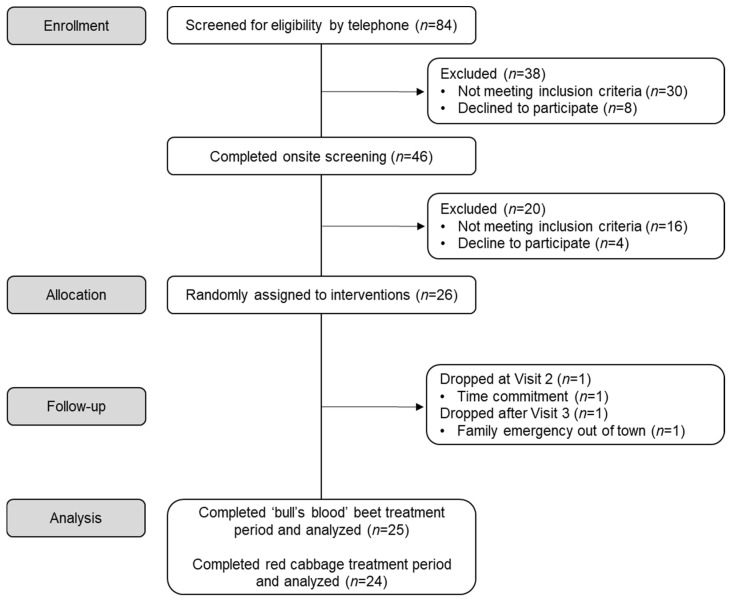
CONSORT flow diagram of participants through a randomized, open-label, 2-period crossover trial where they consumed 2 cups/day of ‘bull’s blood’ beet microgreens or red cabbage microgreens for a 2-week period.

A detailed schematic of participant recruitment and enrollment for the study is provided in Figure 1. A total of 84 individuals responded to advertisements, 46 of which met inclusion criteria through the phone prescreening, were interested in participating in the study, and completed an onsite screening visit. Of those, 26 met inclusion criteria, consented to participate in the study, and were randomly assigned to the intervention. Out of the initial 26 participants, 2 withdrew from the study after randomization and completion of a baseline testing, one participant withdrew following completion of the first treatment period (‘bull’s blood’ beet), while the other withdrew prior to completion of either treatment period. Therefore, data are reported for 25 participants and 24 participants who completed all protocol-specified procedures for the ‘bull’s blood’ beet microgreen treatment and the red cabbage microgreen treatment, respectively. This trial was conducted in accordance with the Declaration of Helsinki, was approved by the CSU Institutional Review Board (19-9119H) and is registered at clinicaltrials.gov as NCT04239898.

### 2.3. Study Design, Randomization, and Intervention

**Figure 2:**
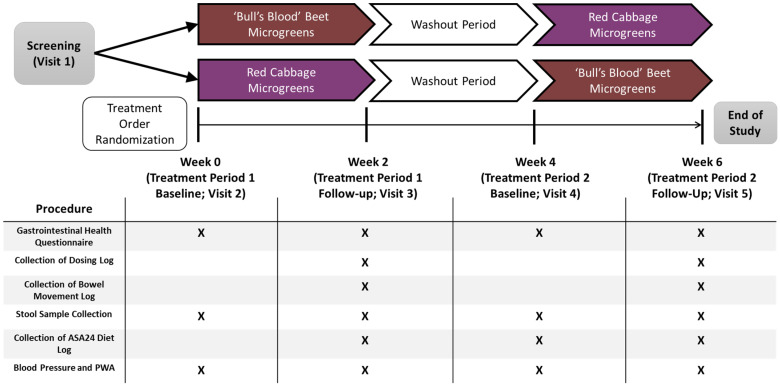
Schematic of clinical trial design and data collection in a randomized, open-label, 2-period crossover trial where they consumed 2 cups/day of ‘bull’s blood’ beet microgreens or red cabbage microgreens for a 2-week period. Procedures completed at each visit are listed with an X. Abbreviations: ASA24, automated self-administered 24-h dietary recall; PWA, pulse wave analysis.

This was a randomized, open-label, 2-period crossover trial in which participants consumed 2 cups daily of (1) ‘bull’s blood’ beet microgreens or (2) red cabbage microgreens for a 2-week period (assigned to intervention in a random order), with each treatment period separated by a 2-week washout period. See Figure 2 for a schematic of the study design. The amount of microgreens used in the intervention was selected based on the standard 2-cup serving size for leafy greens because there is no established serving size for microgreens. A 2-cup serving of each microgreen species was estimated by weighing microgreens in 2-cup amounts 3 different times and calculating the average (30.4 g red cabbage microgreens and 28.1 g ‘bull’s blood’ beet microgreens). Randomization permutations were created using the second generator at Randomization.com (www.randomization.com), and participants were assigned to randomization sequences in order of enrollment into the study.

Participants were provided with a 3–5-day supply of their respective microgreens at a time and were instructed to incorporate the 2-cup regimen into their diets throughout each day rather than eating the full portion at a single sitting. Participants were provided with information about microgreens and how to incorporate them into their diets, as well as a salad spinner with instructions on how to clean microgreens. Participants were asked not to cook the microgreens given there is no published data on how cooking influences their nutritional properties.

### 2.4. Microgreens Production

Microgreens were grown in the CSU Horticulture Center in Fort Collins, Colorado. The specific microgreen species were selected based on previous research evaluating their sensory attributes and consumer acceptability, nutritional characteristics, environmental sustainability, and potential health impacts as well as researcher interest and input regarding their potential for use in reducing disease risk [1,2,3,4,5]. The microgreen species evaluated in the present study belong to the Amaranthaceae and Brassicaceae plant families and included ‘bull’s blood’ beet (*Beta vulgaris*) and red cabbage (*Brassica oleracea var capitate*), respectively. Seeds were purchased from a commercial provider (Johnny’s Selected Seeds, Fairfield, ME, USA). Approximately 1.5 cm of coir fiber was layered in each standard 1020 black polystyrene germination tray (26.7 cm × 53 cm) and seeds were evenly sown at rates of 23 g and 10.5 g per tray, for ‘bull’s blood’ beet and red cabbage, respectively. All trays were covered with black polyethylene sheets for 24–48 h after sowing to increase the germination rate and retain moisture levels. The seeds were grown under light emitting diode lamps (Philips, Andover, MA, USA) for 17 h per day at 62 µmoles·m^2^·s^−1^. Trays were irrigated twice each day with a hand pump sprayer. Sowing of microgreen seeds was staggered to achieve the same growth stage at harvest. Microgreens were harvested 20 days after sowing.

### 2.5. Outcome Measures

Prespecified primary outcome measures to assess feasibility of daily microgreen consumption included participant retention throughout the entire study, and adherence/compliance with the interventions. Prespecified secondary outcome measures included gastrointestinal symptom self-assessment and gastrointestinal health via questionnaires, brachial and aortic blood pressure and augmentation index (i.e., hemodynamics), gut microbiota modulation, and acceptability and feasibility of the interventions through qualitative interviews performed at study completion. Outcomes not prespecified (i.e., exploratory) included evaluation of diet quality during each treatment period relative to the washout period with no microgreen intake (i.e., usual diet).

#### 2.5.1. Compliance and Feasibility

Treatment compliance was assessed by asking study participants to record the dates, times, and amounts of microgreens consumed each day, and to document missing doses and reason for missing the dose (e.g., sick, fell asleep, forgot) in a daily dosing diary. Non-compliance was defined as missing ≥1 full dose (2 cups) per week on average (or <86% compliance).

#### 2.5.2. Gastrointestinal Tolerability

Gastrointestinal tolerability to each microgreen intervention was assessed in 2 ways as previously described [6]. First, a gastrointestinal health questionnaire was completed with study participants at the baseline and 2-week follow-up visits to ascertain perceived gastrointestinal symptoms. Briefly, the questionnaire is separated into 4 sections that assess (A) gastric function, (B) gastrointestinal inflammation, (C) small intestine and pancreas pain, and (D) colon pain. Within each section, participants ranked symptom questions from 0 (no/rarely), 1 (occasionally), 4 (often), to 8 (frequently) and the scores for each question were summed. Gastric function was assessed on a scale of 1 (low priority) to 56 (high priority), gastrointestinal inflammation was assessed from 1 (low priority) to 72 (high priority), intestine and pancreatic pain was assessed from 1 (low priority) to 80 (high priority), and colon pain was assessed as 1 (low priority) to 72 (high priority). Participants that had higher symptom severity scores such that their priority category increased following intervention were classified as “Worsened”. Participants that had lower symptom severity scores such that their priority category decreased following intervention were classified as “Improved”. Participants that did not change between symptom severity categories were classified as “No Change”.

The second way in which gastrointestinal tolerability was assessed was by having participants record bowel movements daily during each 2-week treatment period. Information participants were asked to record included the number of bowel movements, size of bowel movements (small, medium, large), and bowel movement type (Supplemental Table S1). Bowel movement type was based off the Bristol stool chart and was then classified into 3 groups. The Bristol stool chart consists of Type 1 (separate hard lumps), Type 2 (lumpy and sausage like), Type 3 (a sausage shape with cracks in the surface), Type 4 (like a smooth, soft sausage or snake), Type 5 (soft blobs with clear-cut edges), Type 6 (mushy consistency with ragged edges) and Type 7 (liquid consistency with no solid pieces) [7]. Bristol stool types were put into one of three groups, hard (Types 1 and 2), soft (Types 3, 4, or 5), or diarrhea (Type 6 or 7). Stools put into the soft group were considered “normal”, while stools put into the hard and diarrhea group were considered “abnormal”. Stool type was coded as being 1, hard; 2, soft; and 3, diarrhea. Bowel movement size was coded as being 1, small; 2, medium; and 3, large. Bowel movement type, size, and number per day was averaged for day 1, day 2, day 3, first 2 days, first week, and second week of each treatment to evaluate bowel movements for change.

#### 2.5.3. Fecal Collection and Gut Microbiota Analysis

Stool samples were collected at baseline and 2-week follow-up time points for each treatment period and were kept at −20 °C until analysis. DNA was extracted using the FastDNA™ Spin Kit for Feces (MP Biomedicals, Irvine, CA, USA). The V4 region of the 16S rRNA gene was amplified using PCR according to the Earth Microbiome Project protocols using the 515F/806R primers containing a unique 12 bp error-correcting barcode on the forward primer. Amplicon libraries were sequenced on an Illumina Miseq using the MiSeq Reagent Kit v2 (500 cycles) (Illumina, San Diego, CA, USA) at the CSU Next Generation Sequencing Core. All paired-end sequence data were processed using the DADA2 pipeline in QIIME2 (Version 2022.2.0) [8,9,10]. Taxonomy was assigned using the SILVA 138.1 database [11,12,13]. Feature table and taxonomy files were merged and transferred with associated metadata files into MicrobiomeAnalyst marker data profiling [14]. The minimum count filter was set at 4 counts, and the low count filter was set at 10% based on prevalence in samples. Data normalization was performed using total sum scaling. Alpha diversity was assessed using Shannon diversity index, and comparisons analyzed using Mann-Whitney/Kruskal-Wallis tests. Beta diversity was examined using Principal Coordinates Analysis (PCoA) with Bray-Curtis distances analyzed at the genus level using PERMANOVA statistics. Differentially abundant taxa were assessed using a multivariate zero-inflated negative binomial model controlling for sex and age and linear discriminant analysis effect size [14]. Taxa that were significant as measured by both models (*p* < 0.05) are reported as differently abundant.

#### 2.5.4. Blood Collection and Biomarker Analysis

Blood was collected from antecubital veins of participants at baseline and 2-week follow-up time points using standard butterfly needles into vacutainer tubes for separation of serum and plasma and/or immediate analysis of hemoglobin A1c (Alere Afinion Analyzer System, Abbott) and lipid profiles (Piccolo Xpress Chemistry Analyzer, Abbott).

#### 2.5.5. Dietary Intake, Energy Expenditure, and Anthropometric Assessment

Participants completed a 3-day food records (2 weekdays and 1 weekend day) prior to each test visit using the Automated Self-Administered 24-h dietary record (ASA24) and were requested to omit the microgreens from their record [15,16]. ASA24 diet records were also used to calculate Healthy Eating Index-2020 (HEI) score for treatment and washout periods [17,18]. Physical activity patterns were assessed to estimate energy expenditure using a validated 7-day physical activity recall at each visit [19,20]. Height (without shoes) and body weight were assessed using a scale-mounted stadiometer to the nearest 0.5 cm, and a digital scale (Health o Meter Professional, Sunbeam Products, Ibc), respectively. BMI was calculated as weight (kg) divided by height (m^2^). Mid-abdominal waist circumference and hip circumference were measured using a Gulick fiberglass measuring tape with a tension handle (Creative Health Products, Inc., Ann Arbor, MI, USA).

#### 2.5.6. Hemodynamics

Brachial blood pressure was measured, and pulse wave analysis was performed using the Sphygmocor XCEL (AtCor Medical Inc., Naperville, IL, USA) as previously described [21,22]. Briefly, participants rested supine in a climate-controlled, quiet environment for 10 min before brachial systolic (SBP) and diastolic blood pressure (DBP) was measured. Following brachial blood pressure measurement, central aortic blood pressure and other hemodynamic/vascular function-related parameters were measured via pulse wave analysis, including systolic and diastolic pressure, mean arterial pressure (MAP), pulse pressure, heart rate (HR), aortic pressure (AP), augmentation index (AIx), and AIx normalized to a heart rate of 75 bpm (AIx@75).

#### 2.5.7. Food Neophobia, Leafy Green Vegetable Consumption and Intention to Eat Microgreens

At the initial baseline study visit, food neophobia was assessed using the Food Neophobia Scale (FNS), a 10-item questionnaire where participants rated the level to which they agreed or disagreed on a 7-point scale as previously described [4]. High food neophobia is defined as having a score greater than 35, moderate food neophobia is defined as a score between 25 and 35, and a low food neophobia is defined as having a score less than 25 [23]. To assess leafy green vegetable consumption, questions from the NHANES Food Frequency Questionnaire [24] were selected, and response options ranged from “never” to “2 or more times per day” (3 questions). To determine participants prior knowledge of microgreens, microgreen consumption, and intention to purchase microgreens in the future, 5 questions developed for a previous study were utilized [4].

### 2.6. Statistical Analysis

Because this was a feasibility study and previous data regarding microgreen consumption in humans did not exist, a formal power calculation is not recommended and thus was not performed. Data were evaluated for normality with Shapiro-Wilk statistics. If normality was met, data were analyzed using a linear mixed model to compare treatment and time. Non-normal data were converted into log scale for analysis. Participant ID was included in the analyses as a random effect and the analyses were adjusted using the Tukey method. Age and sex were added as covariates in the analyses and retained if significant. Adjusted differences between means are reported along with adjusted *p*-values. SAS v9.4 (SAS Institute Inc., Cary, NC, USA) was used for all statistical analyses except for assessment of diet and energy expenditure which were assessed using R version 4.3.3. Alpha diversity significance was assessed using Kruskal-Wallis, and beta diversity significance was assessed using pairwise PERMANOVA. A *p*-value of 0.05 was used to evaluate statistical significance, and a trend is defined as *p* < 0.10. Descriptive statistics were performed, and all data are represented as mean ± SEM.

## 3. Results

### 3.1. Participant Retention and Baseline Characteristics

The overall attrition rate for the study was 7.7% with 2 of the initial 26 individuals lost to follow-up. Of those, 1 participant withdrew from the study at the baseline visit (visit 2) due to a family emergency. The second participant withdrew from the study after the first follow-up visit (visit 3) due to lack of time; that participant also complained of minor gastrointestinal illness occurring at the beginning of the study but subsequently improved and the participant indicated it was likely due to reasons other than microgreen consumption (i.e., consumption of other foods). Baseline characteristics are presented in Table 1.

### 3.2. Compliance and Feasibility

Compliance with the interventions (Table 2) was 95.6% overall and 96.9% and 94.4% for the ‘bull’s blood’ beet microgreen and red cabbage microgreen interventions, respectively. The average number of days missed (i.e., non-compliant) was less than 1 for each intervention, where the maximum days missed were 3 and 4 for the ‘bull’s blood’ beet and red cabbage interventions, respectively. Participants indicated reasons for non-compliance with microgreen consumption included forgetting, not feeling well/sick, religious purposes (fasting), traveling/out of town.

### 3.3. Gastrointestinal Tolerability

**Figure 3:**
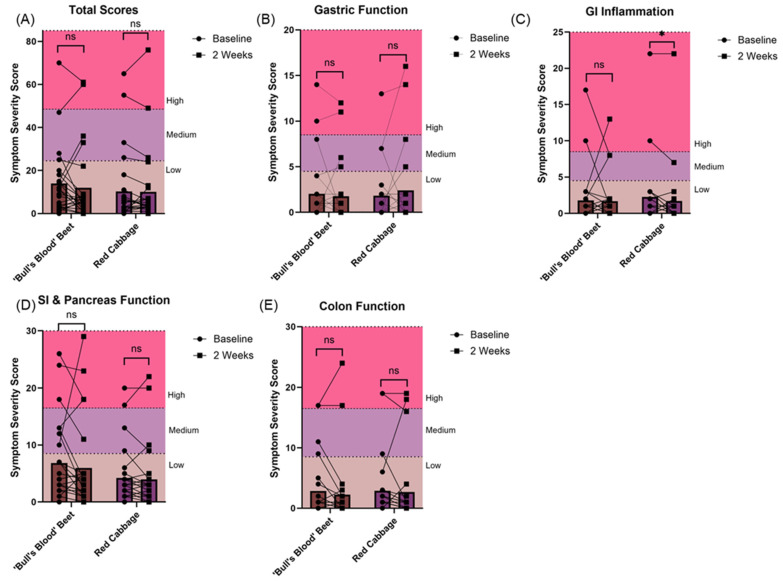
Changes in gastrointestinal symptom severity priority level following daily microgreen consumption as determined by a gastrointestinal health questionnaire in a randomized, open-label, 2-period crossover trial where they consumed 2 cups/day of ‘bull’s blood’ beet microgreens or red cabbage microgreens for a 2-week period. (**A**) total gastrointestinal symptom severity scores, (**B**) gastric function symptom severity scores, (**C**) gastrointestinal inflammation symptom severity scores, (**D**) small intestine and pancreas function symptom severity scores, and (**E**) colon function symptom severity scores. Data are mean ± SEM. * *p* < 0.05. ‘Bull’s Blood’ Beet n = 25; Red Cabbage n = 24. Axes are colored by symptom priority category (low-high). Abbreviations: GI, gastrointestinal; ns, not significant; SI, small intestine.

**Figure 4:**
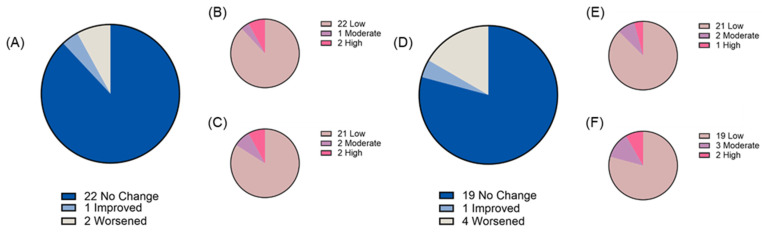
Gastric function symptom severity priority level before and after daily microgreen consumption as determined by a gastrointestinal health questionnaire in a randomized, open-label, 2-period crossover trial where they consumed 2 cups/day of ‘bull’s blood’ beet microgreens or red cabbage microgreens for a 2-week period. Change in priority level for (**A**) ‘bull’s blood’ beet microgreens and number of participants in each priority level at (**B**) baseline and (**C**) 2 weeks following ‘bull’s blood’ beet (n = 25); and change in priority level for (**D**) red cabbage microgreens and number of participants in each priority level at (**E**) baseline and (**F**) 2 weeks following red cabbage (n = 24) microgreens consumption.

**Figure 5:**
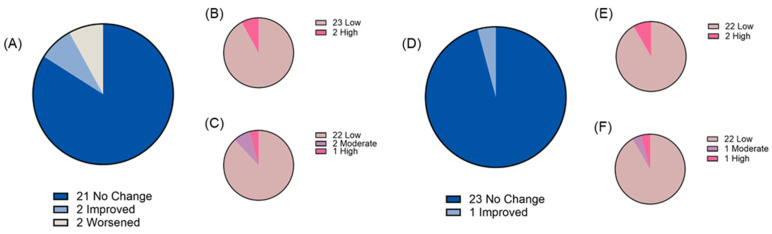
Gastrointestinal inflammation symptom severity priority level before and after daily microgreen consumption as determined by a gastrointestinal health questionnaire in a randomized, open-label, 2-period crossover trial where they consumed 2 cups/day of ‘bull’s blood’ beet microgreens or red cabbage microgreens for a 2-week period. Change in priority level for (**A**) ‘bull’s blood’ beet microgreens and number of participants in each priority level at (**B**) baseline and (**C**) 2 weeks following ‘bull’s blood’ beet (n = 25); and change in priority level for (**D**) red cabbage microgreens and number of participants in each priority level at (**E**) baseline and (**F**) 2 weeks following red cabbage (n = 24) microgreens consumption.

**Figure 6:**
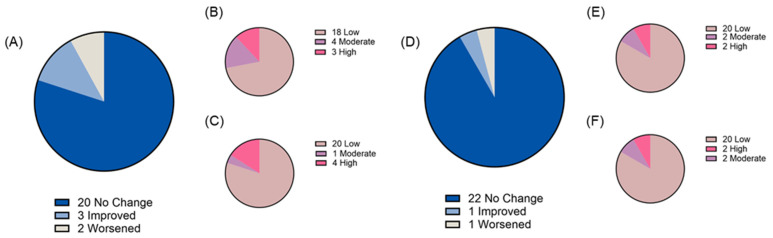
Small intestine and pancreas function symptom severity priority level before and after daily microgreen consumption as determined by a gastrointestinal health questionnaire in a randomized, open-label, 2-period crossover trial where they consumed 2 cups/day of ‘bull’s blood’ beet microgreens or red cabbage microgreens for a 2-week period. Change in priority level for (**A**) ‘bull’s blood’ beet microgreens and number of participants in each priority level at (**B**) baseline and (**C**) 2 weeks following ‘bull’s blood’ beet (n = 25); and change in priority level for (**D**) red cabbage microgreens and number of participants in each priority level at (**E**) baseline and (**F**) 2 weeks following red cabbage (n = 24) microgreens consumption.

**Figure 7:**
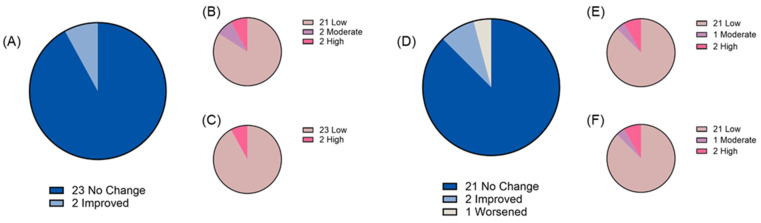
Colon function symptom severity priority level before and after daily microgreen consumption as determined by a gastrointestinal health questionnaire in a randomized, open-label, 2-period crossover trial where they consumed 2 cups/day of ‘bull’s blood’ beet microgreens or red cabbage microgreens for a 2-week period. Change in priority level for (**A**) ‘bull’s blood’ beet microgreens and number of participants in each priority level at (**B**) baseline and (**C**) 2 weeks following ‘bull’s blood’ beet (n = 25); and change in priority level for (**D**) red cabbage microgreens and number of participants in each priority level at (**E**) baseline and (**F**) 2 weeks following red cabbage (n = 24) microgreens consumption.

Two weeks of chronic ‘bull’s blood’ beet microgreen and red cabbage microgreen consumption was found to be tolerable as measured by both gastrointestinal health questionnaire and bowel movement records. At baseline, most participants reported “low” gastrointestinal symptom priority related to gastric function (88% and 87% for ‘bull’s blood’ beet and red cabbage microgreens, respectively), gastrointestinal inflammation (88% and 91% for ‘bull’s blood’ beet and red cabbage microgreens, respectively), small intestine and pancreas pain (69% and 83% for ‘bull’s blood’ beet and red cabbage microgreens, respectively), and colon pain (84% and 87% for ‘bull’s blood’ beet and red cabbage microgreens, respectively). There was no overall difference in total symptom severity score following intervention observed; however, there was a slight but significant (*p* = 0.047) improvement in gastrointestinal inflammation symptom severity scores following red cabbage microgreen intervention (Figure 3A–E). On an individual basis, some participants experienced changes in their symptom priority category (low, medium, and high; Figure 4, Figure 5, Figure 6 and Figure 7). The participants who worsened following interventions consisted of the same 6 individuals that worsened across multiple symptom categories.

**Figure 8:**
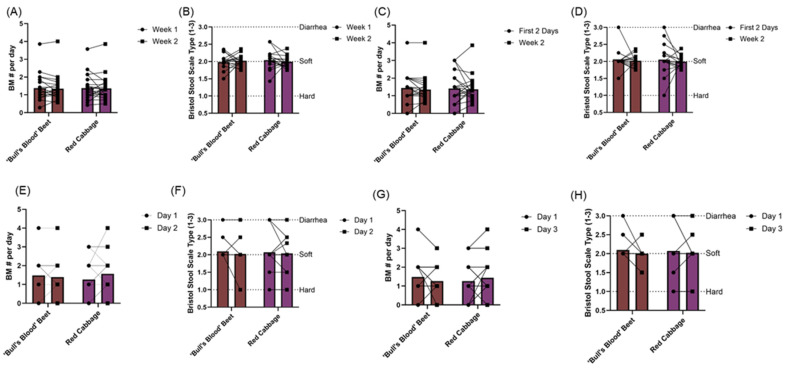
Average bowel movement frequency and type was evaluated through a bowel movement log (i.e., Bristol stool scale) in a randomized, open-label, 2-period crossover trial where they consumed 2 cups/day of ‘bull’s blood’ beet microgreens or red cabbage microgreens for a 2-week period. (**A**) Number of bowel movements per day and (**B**) Bristol stool scale bowel movement type comparisons between the first vs. second week of microgreens intervention, (**C**) number of bowel movements per day and (**D**) Bristol stool scale bowel movement type comparisons between the first two days vs. the second week of intervention, (**E**) number of bowel movements per day and (**F**) Bristol stool scale bowel movement type comparisons between day 1 vs. day 2 of intervention, (**G**) number of bowel movements per day and (**H**) Bristol stool scale BM type comparisons between the day 1 vs. day 3 of the intervention. Data are mean ± SEM. ‘Bull’s Blood’ Beet n = 25; Red Cabbage n = 24. Participants with identical bowel movement data overlap and appear as one point. Abbreviations: BM, bowel movement.

To further evaluate the gastrointestinal tolerability of daily microgreens consumption, participants recorded the Bristol stool scale type for each bowel movement in a daily log. On average, participants had “normal” stool consistency and produced between 1–2 bowel movements per day. There were no differences in bowel movement type or frequency between the beginning and the end of either intervention (Figure 8A–H).

### 3.4. Gut Microbiota

**Figure 9:**
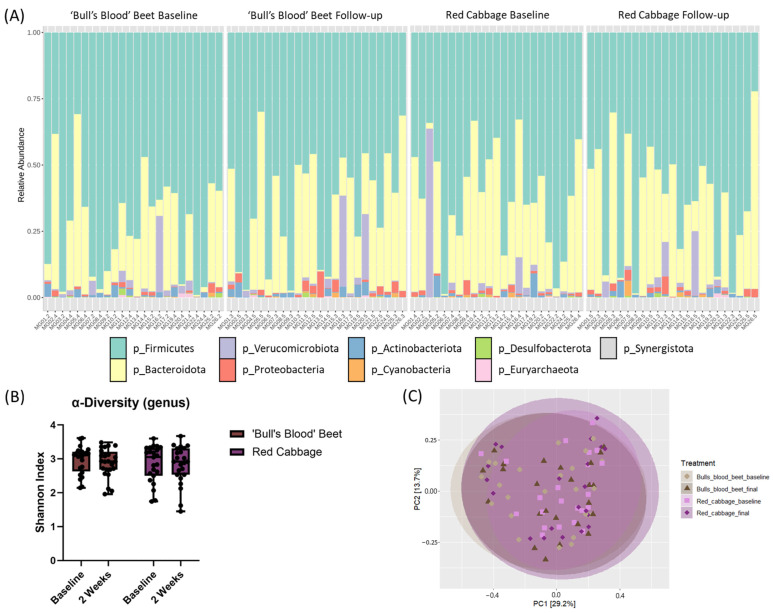
Gut microbiota parameters at baseline and 2-weeks following daily consumption of 2 cups/day of ‘bull’s blood’ beet microgreens or red cabbage microgreens in a randomized, open-label, 2-period crossover trial. (**A**) Relative abundance of taxa at the phylum level, (**B**) alpha diversity of the gut microbiota at the genus level measured by Shannon index, and (**C**) beta diversity of the gut microbiota PCoA plots at the genus level measured by Bray-Curtis. ‘Bull’s blood’ beet n = 24; red cabbage n = 23.

To evaluate the effects of daily microgreen consumption on the gut microbiota, the relative abundance of taxa was measured using 16S rRNA sequencing (Figure 9A). There were no significant differences in alpha diversity or beta diversity (Figure 9B,C) or in relative abundance at the phylum or genus level following 2 weeks of daily microgreen consumption or between groups.

### 3.5. Anthropometrics, Energy Expenditure, and Dietary Intake

There were no significant differences (*p* > 0.05) in body weight, BMI, waist circumference, hip circumference, and waist-to-hip ratio following 2 weeks of daily microgreen consumption (Table 3). Additionally, average daily energy expenditure calculated from a 7-day physical activity recall (Table 4) indicated participants expended an average of 2536 kcal and 38.3 METs at baseline and 2318 kcal and 35.2 METs at 2 weeks (*p* = 0.012 and *p* = 0.013 vs. baseline, respectively) for the ‘bull’s blood’ beet microgreen treatment period and 2429 kcal and 36.9 METS at baseline and 2398 kcal and 36.5 METs at 2 weeks (*p* = 0.915 and *p* = 0.970 vs. baseline, respectively) for the red cabbage microgreen treatment period. Analysis of selected dietary components is presented in Supplemental Table S2.

When diet was evaluated using ASA24, there were no significant differences in kcal consumed over the 2-week treatment periods or between groups; however, the red cabbage microgreen intervention period had increased HEI scores (Table 5) compared to the washout period that trended towards significance (*p* = 0.084), though there were no differences relative to the ‘bull’s blood’ beet microgreen treatment period. The average HEI scores for each phase were 66, 72, and 64 for the ‘bull’s blood’ beet, red cabbage, and washout phases, respectively, and when all phases are averaged, the HEI score was 68 overall. When components of the HEI score were compared between periods, participants had improved saturated fat HEI component scores while consuming microgreens compared to the washout period (*p* = 0.006 and *p* = 0.030 for ‘bull’s blood’ beet and red cabbage microgreens, respectively). Participants also had better total vegetable HEI component scores (*p* = 0.056) during the red cabbage microgreen period compared to the washout period, as well as better sodium HEI component scores during the red cabbage microgreens treatment period compared to the ‘bull’s blood’ beet microgreens treatment period (*p* = 0.036). Lastly, participants had lower scores for added sugar during the ‘bull’s blood’ beet intervention than the red cabbage and washout periods (*p* = 0.070 and *p* = 0.084, respectively).

### 3.6. Hemodynamics

There were no significant differences in brachial or central aortic blood pressure, HR, MAP, AP, AIx or AIx@75 following 2 weeks of daily ‘bull’s blood’ beet or red cabbage microgreen consumption and there were no differences between groups (Table 6).

### 3.7. Baseline Dietary Habits

At baseline, participants were asked about their typical dietary habits regarding leafy green vegetable consumption, knowledge and consumption of microgreens, and willingness to try new foods (Table 7). On average, participants consumed raw greens 1–2 times per week and were “a little” to “somewhat” familiar with microgreens. Participants reported “rarely” consuming microgreens but indicated a “slight likelihood” of purchasing microgreens in the future. Additionally, participants on average were classified as having low food neophobia.

## 4. Discussion

Microgreens are a novel horticultural food crop gaining popularity amongst consumers. Their rich nutrient and phytochemical profiles, organoleptic properties, and implications for environmental sustainability make them a promising functional food for promoting human health and attenuating chronic disease with aging. To our knowledge, this is the first study to explore the feasibility, tolerability, and potential health effects of daily ‘bull’s blood’ red beet microgreen and red cabbage microgreen consumption in MA/O humans. In this study, high participant retention and intervention compliance supports the feasibility of incorporating these microgreens into the daily diet. Furthermore, we found that daily consumption of 2 cups of fresh/raw ‘bull’s blood’ beet microgreens or red cabbage microgreens for 2 weeks did not have major adverse impacts on gastrointestinal symptom severity or bowel movement patterns indicating gastrointestinal tolerability of daily microgreen consumption in this study population.

While most participants experienced no change or improved gastrointestinal symptom severity with daily microgreen consumption, there were 6 participants who experienced a worsening of symptoms in 1 or more categories outlined in our questionnaire (2 following ‘bull’s blood’ beet microgreen intervention, 2 following red cabbage microgreens intervention, and 2 following both interventions). 5 out of these 6 participants had symptom severity scores above the “low” category at baseline. In contrast, 1 participant had “low” gastric function symptom severity prior to red cabbage and ‘bull’s blood’ beet microgreen consumption and rose to “moderate” after 2 weeks of chronic consumption. These changes in symptom severity may be a result of increased fiber in the diet from the microgreens as well as other microbiota-accessible phytochemicals like anthocyanins. Interestingly, while these individuals showed worsened symptoms in some categories, improvements in symptom severity in other categories were not uncommon. For example, 1 participant worsened in gastric function, small intestine and pancreas pain, and colon pain categories following red cabbage microgreen intervention, but improved in colon symptom severity following ‘bull’s blood’ beet intervention. It is possible that there may be a relationship between the baseline diet and responses to the microgreen interventions; however, diet records before starting any intervention were not collected which is a limitation of the present study. Furthermore, the low number of individuals that experienced worsened symptoms makes the statistical testing underpowered. It is also possible that the worsening in symptoms is related to another lifestyle and/or biological factor that was not captured in our data collection. Importantly, these detected changes in gastrointestinal symptoms did not lead to individual complaints by study participants to research staff, reporting of adverse events, or withdrawal from the study. Furthermore, there were no significant changes in bowel movement frequency or quality over the course of the intervention; however, records were not kept during the washout periods of no microgreen consumption which is a limitation. Overall, these data and information suggest that daily microgreen consumption is tolerated by the gastrointestinal tract but that some individuals may have minor sensitivity. Future research is needed to understand gastrointestinal health impacts of daily microgreen consumption in humans.

We also found that incorporation of specific microgreens into daily diets may also improve the quality of the rest of the diet, particularly through increased total vegetable and reduced saturated fat consumption. In participant interviews and dosing logs, we found that common methods of microgreens consumption included smoothies, sandwiches, and leafy green salads which could be contributing to the differences in diet quality between the red cabbage microgreen and washout periods. This improvement in diet quality can also contribute to a reduction in chronic disease risk since diet is a major modifiable risk factor. Additionally, we found that sodium and added sugar HEI component scores were better in the red cabbage microgreens treatment period compared to the ‘bull’s blood’ beet microgreens treatment period. This may be attributed to the lower mean liking score for flavor associated with the ‘bull’s blood beet microgreens compared to the red cabbage microgreens that our group previously reported [4]; however, this requires confirmation. In interviews with participants at the conclusion of the study, many participants reported more bitterness associated with the ‘bull’s blood’ beet microgreens. This could have led participants to increase their salt and added sugar consumption to mask undesirable flavors from this species of microgreens. Despite a reduction in the sodium and added sugar component score, the average overall HEI score of the participants is still higher than the national average of 58 [17]. Thus, it is unclear how these findings will translate to populations with lower baseline diet quality. A HEI score of 100 indicates complete adherence to the Dietary Guidelines for Americans, so a score of 68 indicates 68% adherence. One major limitation of the study is low diet record compliance, specifically during the washout period where only about half of participants completed records during that period. Another limitation is the lack of baseline diet records for comparison with intervention periods, therefore comparisons were made with the washout period instead. Whether daily microgreen consumption can improve overall diet quality requires further evaluation.

Microgreens have been demonstrated to be rich in nutrients and phytochemicals with demonstrated cardiovascular-protective effects. For instance, red beets and red beet microgreens are rich in nitrate which can impact cardiovascular function through ultimate conversion to the vasodilatory and vascular-protective molecule nitric oxide [25,26]. Nitric oxide is essential for maintenance of vascular tone and function, and regulation of blood pressure, and dietary sources of nitrate have been shown to improve these parameters in certain populations, such as those with hypertension [27]. Betalains are phytochemicals found in red beets and red beet microgreens, as well as other foods such as dragon fruit, that have been shown to have beneficial impacts on vascular function postprandially [26,28]. Additionally, red cabbage microgreens are rich in polyphenols including anthocyanins, as well as glucosinolates, which have also been shown to have cardiovascular-protective effects including benefits to blood pressure [2,29,30]. As expected, our study found that 2 weeks of daily consumption of ‘bull’s blood’ beet microgreens or red cabbage microgreens did not impact brachial and aortic hemodynamics of healthy MA/O adults. This is likely due in large part to the short duration of the study, the health status of study participants, and the feasibility nature of the clinical trial. Additionally, we did not evaluate the phytochemical concentrations in the microgreens used in the present study, which is a limitation. Because the cardiovascular effects of microgreens were secondary outcomes in the present study, the study was not designed or powered to evaluate potential benefits. Additionally, the participants in the study were generally healthy with healthy blood pressure and vascular function, so changes in these measures would not necessarily be feasible or beneficial. To investigate if there are beneficial effects of these foods on these and other measures of cardiometabolic health, acute postprandial studies and longer chronic intervention clinical trials designed to evaluate these as primary outcomes are needed. Additionally, future studies should characterize the phytochemical and nutrient concentrations in the microgreens evaluated to understand potential impacts on cardiovascular health and function and provide insight into dosing.

The impact of daily ‘bull’s blood’ beet microgreen and red cabbage microgreen consumption on the gut microbiota was explored. Red beet and red cabbage microgreens contain phytochemicals that can exert prebiotic-like effects such as polyphenols, and those such as polyphenols and glucosinolates that are metabolized by the gut microbiota to bioactive metabolites that enter circulation [31,32]. Additionally, due to the novelty of these food crops and unknown effects on gastrointestinal health, we considered the possibility that there may be adverse effects on the gut microbiota as well. There were no significant differences found in the alpha diversity or beta diversity of the gut microbiota following the interventions. Additionally, there were no differences in the abundance of different gut microbial taxa. In contrast, research in mice showed that 8 weeks of red cabbage microgreens supplementation increased alpha and beta diversity and altered the relative abundance of specific taxa; however, that study was performed in a high fat diet-fed rodent model and employed a longer intervention [3]. Research evaluating the potential human health impacts of microgreens is limited and though not an equal comparator, research with sprouts may provide insight into the potential health effects of microgreens. In humans, the production of beneficial glucosinolate metabolites following sprout consumption is associated with specific microbial taxa like *Bifidobacterium* [33]. Additionally, it is known that consumption of foods rich in phytochemicals like anthocyanins and glucosinolates can modulate the gut microbiome [34,35]. Resilience of the gut microbiota composition supports the gastrointestinal tolerability of chronic microgreen consumption; however, further research with longer intervention durations is necessary to fully elucidate the relationship between the gut microbiome and chronic daily microgreen consumption [36,37]. Additionally, the mediating role of baseline gut microbiome composition in determining postprandial and chronic intervention responses (e.g., circulating phytochemical metabolites, physiological outcomes) should be evaluated.

## 5. Conclusions

This feasibility study was the first to explore the gastrointestinal tolerability and potential health effects of ‘bull’s blood’ beet and red cabbage microgreens in humans and provides a foundation for future clinical research involving these foods. Overall, we found that daily consumption of ‘bull’s blood’ beet and red cabbage microgreens is feasible and tolerable in healthy MA/O adults. Preclinical research suggests that chronic red cabbage microgreen consumption has positive effects on blood lipids and can modulate the gut microbiome [2,3]. Clinical evidence suggests that the glucosinolate glucoraphanin and its metabolite sulforaphane is bioavailable from broccoli microgreens [38]. Red cabbage microgreens are rich in glucosinolates which have been shown to have cardiovascular-protective, anti-cancer, and anti-inflammatory properties, supporting their use as a functional food [39,40,41]. They also contain anthocyanins which have been linked to improved human health (e.g., cardiometabolic, cognitive/brain) [42]. Importantly, red cabbage microgreen anthocyanin contents can be enhanced through manipulation of the controlled environment during production (e.g., lighting, temperature) which may augment health effects [43]. Future research should evaluate manipulation of the controlled environment for increasing anthocyanin biosynthesis and whether subsequent increases in anthocyanin contents translate to improved human health effects. Such research should be coupled with evaluation of impacts on other nutrients and phytochemicals, consumer acceptability, and plant quality characteristics. Though red beet microgreens have not been previously evaluated in terms of their human health effects, mature red beet consumption has been linked to improved cardiovascular function and reduced CVD risk, and red beet microgreens are rich in many of the same health-promoting bioactive compounds [22,44,45]. Overall, ‘bull’s blood’ beet and red cabbage microgreens are promising nutrient- and phytochemical-rich food crops for increasing sustainability and accessibility in the food system and for addressing chronic diseases associated with aging such as CVD. Limitations to this study not previously mentioned include the lack of a run-in washout period prior to the start of the study to minimize effects of the background diet on outcomes, the short 2-week intervention period, and lack of background diet standardization. However, because the primary objective of this study was to evaluate the feasibility and gastrointestinal tolerability of daily microgreen consumption within a usual dietary pattern, manipulation of the background diet was not appropriate. Future studies focused on bioavailability of phytochemicals and/or nutrients in microgreens and health effects associated with those compounds should consider a washout period prior to study start to minimize background diet effects, as well as control of the background diet throughout the course of the study. Future human studies should focus on the health impacts of these foods in diverse human populations over longer intervention periods, including bioavailability of nutrients and phytochemicals from various microgreens, dose responses, and physiological, health, and disease related outcomes.

## Figures and Tables

**Table 1:** Screening characteristics of participants who completed a randomized, open-label, 2-period crossover trial where they consumed 2 cups/day of ‘bull’s blood’ beet microgreens or red cabbage microgreens for a 2-week period (n = 24). Values are mean ± SEM. Abbreviations: BMI, body mass index; DBP, diastolic blood pressure; HDL, high-density lipoprotein; LDL, low-density lipoprotein; SBP, systolic blood pressure.

	Completed (n = 24)
Age, years	57 ± 1
Years postmenopausal (women only)	8 ± 1
Sex, M:F (n)	9:15
BMI, kg/m^2^	23.7 ± 0.5
Waist-to-hip ratio	0.84 ± 0.02
Total cholesterol, mg/dL	200 ± 4
HDL, mg/dL	71 ± 3
LDL, mg/dL	110 ± 4
Triglycerides, mg/dL	88 ± 6
HDL:LDL ratio	0.67 ± 0.04
Hemoglobin A1c, %	5.3 ± 0.1
SBP, mmHg	110 ± 3
DBP, mmHg	69 ± 2

**Table 2:** Overall compliance and compliance with each microgreen intervention in a randomized, open-label, 2-period crossover trial where they consumed 2 cups/day of ‘bull’s blood’ beet microgreens or red cabbage microgreens for a 2-week period.

	Days Compliant	Days Non-Compliant	Percent Compliance
Overall	13.4 ± 0.2	0.6 ± 0.2	95.6 ± 1.5
‘Bull’s Blood’ Beet	13.6 ± 0.2	0.4 ± 0.2	96.9 ± 1.2
Red Cabbage	13.2 ± 0.2	0.8 ± 0.2	94.4 ± 1.6

Values are mean ± SEM. ‘Bull’s Blood’ Beet n = 25; Red Cabbage n = 24.

**Table 3:** Anthropometric parameters at baseline and 2-weeks following daily consumption of 2 cups/day of ‘bull’s blood’ beet microgreens or red cabbage microgreens in a randomized, open-label, 2-period crossover trial.

	‘Bull’s Blood’ Beet		Red Cabbage	
	Baseline	2 Weeks	Baseline	2 Weeks
Body weight, kg	65.4 ± 1.8	65.6 ± 1.8	66.2 ± 2.0	65.9 ± 2.0
BMI, kg/m^2^	23.6 ± 0.5	23.7 ± 0.5	23.8 ± 0.5	23.7 ± 0.5
WC, cm	81 ± 2	81 ± 2	80 ± 2	82 ± 2
HC, cm	97 ± 1	97 ± 1	97 ± 1	98 ± 1
WC:HC	0.84 ± 0.01	0.83 ± 0.01	0.83 ± 0.02	0.84 ± 0.02

Values are mean ± SEM. ‘Bull’s Blood’ Beet n = 25; Red Cabbage n = 24. Abbreviations: BMI, body mass index; WC, waist circumference; HC, hip circumference.

**Table 4:** Energy expenditure at baseline and 2-weeks following daily consumption of 2 cups/day of ‘bull’s blood’ beet microgreens or red cabbage microgreens in a randomized, open-label, 2-period crossover trial.

	‘Bull’s Blood’ Beet		Red Cabbage	
	Baseline	2 Weeks	Baseline	2 Weeks
Average daily energy expenditure, Kcal	2536 ± 137	2318 ± 79 *	2430 ± 68	2399 ± 76
Average daily METs	38.3 ± 1.2	35.2 ± 0.4 *	36.9 ± 0.6	36.5 ± 0.7

Values are mean ± SEM. Abbreviations: METs, metabolic equivalents. * *p* < 0.05 compared to baseline. n = 24.

**Table 5:** Healthy Eating Index for each treatment period in a randomized, open-label, 2-period crossover trial where participants consumed 2 cups/day of ‘bull’s blood’ beet microgreens or red cabbage microgreens for 2 weeks, separated by a 2-week washout period.

	‘Bull’s Blood’ Beet	Red Cabbage	Washout
Score Range: 0–5			
Total Vegetables	4.7 ± 0.2 ^a#^	4.9 ± 0.1 ^a^	4.4 ± 0.4 ^a^
Greens and Beans	3.6 ± 0.4 ^a^	3.1 ± 0.4 ^a^	3.1 ± 0.7 ^a^
Total Fruit	3.2 ± 0.4 ^a^	3.7 ± 0.3 ^a^	3.4 ± 0.7 ^a^
Whole Fruit	3.8 ± 0.4 ^a^	4.2 ± 0.3 ^a^	3.7 ± 0.6 ^a^
Total Protein	4.2 ± 0.3 ^a^	4.6 ± 0.2 ^a^	4.2 ± 0.4 ^a^
Seafood and Plant Protein	3.9 ± 0.4 ^a^	4.5 ± 0.2 ^a^	3.7 ± 0.6 ^a^
Score Range: 0–10			
Whole Grain	3.8 ± 0.6 ^a^	5.7 ± 0.7 ^a^	4.5 ± 1.1 ^a^
Total Dairy	5.4 ± 0.6 ^a^	4.7 ± 0.5 ^a^	5.6 ± 1.0 ^a^
Fatty Acids	7.6 ± 0.5 ^a^	7.9 ± 0.5 ^a^	6.2 ± 0.9 ^a^
Sodium	2.9 ± 0.7 ^a^	4.8 ± 0.7 ^b^	4.4 ± 0.9 ^ab^
Refined Grain	7.7 ± 0.7 ^a^	8.4 ± 0.5 ^a^	7.4 ± 1.1 ^a^
Saturated Fat	7.6 ± 0.4 ^a^	6.8 ± 0.5 ^a^	5.2 ± 1.0 ^b^
Added Sugar	7.8 ± 0.4 ^a#%^	8.7 ± 0.3 ^a^	9.1 ± 0.4 ^a^
Total	66.2 ± 2.2 ^a^	72.0 ± 2.0 ^a#^	64.9 ± 3.4 ^a^

Values are mean ± SEM, n = 25 for bull’s blood beet, n = 24 for red cabbage, n = 9 for washout. HEI score components are on 0–5 range or 0–10 range, where higher scores indicate closer adherence to the Dietary Guidelines for Americans. Different letters indicate statistically significant differences (*p* < 0.05). ^#^ *p* < 0.10 vs. washout. ^%^ *p* < 0.10 vs. red cabbage microgreens.

**Table 6:** Hemodynamics at baseline and 2-weeks following daily consumption of 2 cups/day of ‘bull’s blood’ beet microgreens or red cabbage microgreens in a randomized, open-label, 2-period crossover trial.

	‘Bull’s Blood’ Beet		Red Cabbage	
	Baseline	2 Weeks	Baseline	2 Weeks
Brachial SBP, mmHg	118 ± 2	117 ± 2	118 ± 2	118 ± 3
Brachial DBP, mmHg	71 ± 2	70 ± 2	73 ± 1	72 ± 2
Aortic SBP, mmHg	107 ± 2	106 ± 2	109 ± 2	109 ± 3
Aortic DBP, mmHg	72 ± 2	71 ± 2	73 ± 1	72 ± 2
HR, bpm	56 ± 2	56 ± 2	55 ± 2	57 ± 2
MAP, mmHg	84 ± 2	83 ± 2	86 ± 2	85 ± 2
AP, mmHg	12 ± 3	9 ± 1	9 ± 1	13 ± 3
AIx, %	24 ± 2	25 ± 3	25 ± 3	28 ± 3
AIx@75, %	16 ± 3	17 ± 3	16 ± 3	20 ± 4

Values are mean ± SEM, n = 25 for bull’s blood beet and n = 24 for red cabbage. Abbreviations: SBP, systolic blood pressure; DBP, diastolic blood pressure; HR, heart rate; MAP, mean arterial pressure; AP, aortic pressure; AIx, augmentation index; AIx@75, augmentation index normalized to 75 beats per minute.

**Table 7:** Food neophobia, leafy green vegetable consumption, microgreen knowledge, and microgreen consumption at baseline in a randomized, open-label, 2-period crossover trial where participants consumed 2 cups/day of ‘bull’s blood’ beet microgreens or red cabbage microgreens for 2 weeks.

	Completed (n = 24)
Food neophobia score	21.3 ± 1.3
How often did you eat raw greens (such as spinach, turnip, collard, mustard, chard, or kale)?	6.2 ± 0.5
How often did you eat lettuce salads (with or without other vegetables?	7.1 ± 0.4
How often were the lettuce salads you ate made with dark green leaves?	7.2 ± 0.3
How familiar are you with microgreens?	2.6 ± 0.2
How frequently have you consumed microgreens?	2.1 ± 0.2
How likely are you to purchase microgreens in the future?	4.7 ± 0.3

Data are mean ± SEM. n = 24. There were no significant differences. Leafy green vegetable consumption: 1 = never, 2 = 1–6 times per year, 3 = 7–11 times per year, 4 = 1 time per month, 5 = 2–3 times per month, 6 = 1 time per week, 7 = 2 times per week, 8 = 3–4 times per week, 9 = 1 time per day, 10 = 2 or more times per day. Familiarity with microgreens: 1 = not at all familiar, 2 = a little familiar, 3 = somewhat familiar, 4 = familiar, 5 = very familiar. Frequency of microgreen consumption: 1 = never, 2 = rarely, 3 = sometimes, 4 = often. Intention to purchase microgreens: 1 = extremely unlikely, 2 = unlikely, 3 = more or less unlikely, 4 = neutral, 5 = more or less likely, 6 = very likely, 7 = extremely likely.

## Data Availability

The original contributions presented in this study are included in the article. Further inquiries can be directed to the corresponding author.

## Supplementary Materials

The following supporting information can be downloaded at: https://www.mdpi.com/article/10.3390/nu17030467/s1, Table S1: Bowel Movement Daily Monitoring Record; Table S2: Macronutrient and Micronutrient Intake.
