# Supplementary material for: Feasibility and Tolerability of Daily Microgreen Consumption in Healthy Middle-Aged/Older Adults: A Randomized, Open-Label, Controlled Crossover Trial
Source: Nutrients. 2025 Jan 28;17(3):467. doi: 10.3390/nu17030467 (PMC11820112; doi:10.3390/nu17030467)
Supplement: Supplementary file 1 [file nutrients-17-00467-s001.zip › nutrients-3397048-supplementary.pdf]

Table S1: Bowel Movement Daily Monitoring Record

BOWEL MOVEMENT (B.M.) - DAILY MONITORING RECORD

When recording B.M.'s, note both **SIZE:** L = Large M = Medium SM = Small e.g., Large soft stool =
and **TYPE :** H = Hard S = Soft D = Diarrhea
(for TYPE, numbered 1 to 7, you can also use the Bristol Stool Chart on back of page)

L S	L 3	L 4

X = no B.M.

DATE	1	2	3	4	5	6	7	8	9	10	11	12	13	14	15	16	17	18	19	20	21
1st Stool																					
2nd Stool																					
3rd Stool																					
4th Stool																					
DATE	23	24	25	26	27	28	29	30	31	32	33	34	35	36	37	38	39	40	41	42	43
1st Stool																					
2nd Stool																					
3rd Stool																					
4th Stool																					

Notes: ____ **SEE OTHER SIDE**

Table S1: Bowel Movement Daily Monitoring Record

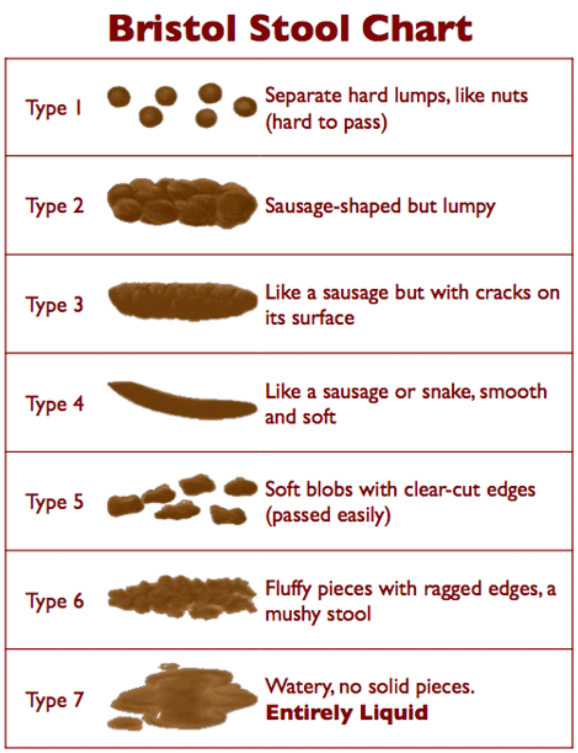

Types **1** and **2** indicate constipation (Hard)
Types **3** and **4** are the easiest to pass (Soft)
Types **5 - 7** may indicate Diarrhea

Reference:

Lewis SJ. Heaton KW. Stool form scale as a useful guide to intestinal transit time. *Scandinavian Journal of Gastroenterology* 1997; 32(9): 920-4.

Table S2: Macronutrient and Micronutrient intake

	'Bull's Blood' Beet	Red Cabbage	Washout
Nutrients			
Protein (g)	74.4 ± 5.6 (31.8 - 121.5)[a]	73.5 ± 5.5 (27.2 - 117.6)[a]	68.5 ± 12.7 (33.3 - 158.2)[a]
Carbohydrates (g)	219.6 ± 14.3 (99.5 -367.4)[a]	214.9 ± 14.4 (96.7 - 373.5)[a]	174.7 ± 23.8 (100.2 - 321.0)[a]
Fat (g)	75.1 ± 6.9 (28.8 - 177.9)[a]	78.9 ± 6.9 (32.4 - 147.1)[a]	74.4 ± 11.9 (12.9 - 123.6)[a]
Fiber (g)	25.8 ± 2.5 (10.2 - 63.2)[a]	26.6 ± 2.1 (9.4 - 49.2)[a#]	21.1 ± 3.4 (5.9 - 41.9)[a]
Vitamin C (mg)	88.3 ± 10.3 (15.5 - 199.6)[a]	134.1 ± 10.6 (46.3 - 231.8)[b]	90.3 ± 22.8 (3.7 - 202.0)[ab]
Vitamin A (µg)	668.4 ± 66.9 (265.3 - 1595.6)[a]	755.0 ± 97.6 (203.3 - 1812.8)[a]	630.5 ± 98.6 (268.6 - 1270.7)[a]
Vitamin E (mg)	11.2 ± 1.7 (3.5 - 40.1)[a]	12.6 ± 1.9 (4.3 - 35.5)[a#]	8.1 ± 1.4 (2.8 - 19.9)[a]
Vitamin K (µg)	264.6 ± 66.1 (28.8 - 1498.2)[a]	252.8 ± 37.5 (46.5 - 736.3)[a]	187.2 ± 36.7 (32.8 - 312.0)[a]
Calcium (mg)	862.3 ± 75.6 (329.4 - 1482.8)[a]	848.5 ± 78.6 (337.4 - 1575.4)[a]	769.6 ± 156.0 (326.2 - 1917.6)[a]
Iron (mg)	13.4 ± 1.0 (7.7 - 26.8)[a]	15.0 ± 1.7 (6.7 - 39.3)[a#]	10.7 ± 1.5 (4.7 - 17.6)[a]
Potassium (mg)	3113.5 ± 207.0 (1465.5 - 4869.6)[a]	3131 ± 195.2 (1606.0 - 5090.7)[a]	2575.5 ± 383.0 (1328.3 - 5207.2)[b]
Sodium (mg)	3231.8 ± 161.9 (2017.2 - 4290.8)[a]	2956.9 ± 228.4 (1218.0 - 5223.5)[a]	2540.5 ± 373.6 (1220.4 - 4449.8)[a]
Magnesium (mg)	364.8 ± 37.5 (166.0 - 964.8)[a]	362.3 ± 30.1 (171.9 - 807.3)[a]	290.7 ± 39.7 (141.3 - 554.3)[a]

Macronutrient and micronutrient intake during treatment and washout periods. Values are mean ± SEM (ranges). Different letters indicate statistically significant differences. # p<0.10 vs washout.
